# P-, but not E- or L-, selectin-mediated rolling adhesion persistence in hemodynamic flow diverges between metastatic and leukocytic cells

**DOI:** 10.18632/oncotarget.18786

**Published:** 2017-06-28

**Authors:** Erin Elizabeth Edwards, Jaeho Oh, Ananyaveena Anilkumar, Katherine Gayle Birmingham, Susan Napier Thomas

**Affiliations:** ^1^ Wallace H. Coulter Department of Biomedical Engineering, Georgia Institute of Technology and Emory University, Atlanta, Georgia, USA; ^2^ Parker H. Petit Institute for Bioengineering and Bioscience, Georgia Institute of Technology, Atlanta, Georgia, USA; ^3^ George W. Woodruff School of Mechanical Engineering, Georgia Institute of Technology, Atlanta, Georgia, USA; ^4^ Winship Cancer Institute, Emory University, Atlanta, Georgia, USA

**Keywords:** metastasis, leukocyte, microfluidic, shear stress, heparin

## Abstract

The ability of leukocytic cells to engage selectins via rolling adhesion is critical to inflammation, but selectins are also implicated in mediating metastatic dissemination. Using a microfluidic- and flow-based cell adhesion chromatography experimental and analytical technique, we interrogated the cell-subtype differences in engagement and sustainment of rolling adhesion on P-, E-, and L-selectin-functionalized surfaces in physiological flow. Our results indicate that, particularly at low concentrations of P-selectin, metastatic but not leukocytic cells exhibit reduced rolling adhesion persistence, whereas both cell subtypes exhibited reduced persistence on L-selectin and high persistence on E-selectin, differences not revealed by flow cytometry analysis or reflected in the extent or velocity of rolling adhesion. Conditions under which adhesion persistence was found to be significantly reduced corresponded to those exhibiting the greatest sensitivity to a selectin-antagonist. Our results suggest that potentially therapeutically exploitable differences in metastatic and leukocytic cell subtype interactions with selectins in physiological flow are identifiable through implementation of functional assays of adhesion persistence in hemodynamic flow utilizing this integrated, flow-based cell adhesion chromatography analytical technique.

## INTRODUCTION

Circulating cell recruitment is critical to a variety of physiological and pathophysiological processes and occurs amidst the high shear environment of the vasculature via a multistep rolling to firm adhesion cascade [1]. Cells initially engage with the vascular endothelium via rolling adhesion through fast kinetic interactions between endothelial-presented selectins and their corresponding circulating cell-presented ligands. Selectins, which include P-, E-, and L-selectin that differ structurally in the number of consensus repeats that each contains, exhibit distinct profiles of cell and tissue expression that presumably underlie their functional roles in cell homing [1]. E-selectin is primarily expressed on endothelial cells [2], and mediates slow, steady rolling adhesion [3], whereas L-selectin is constitutively expressed on leukocytes [4] and facilitates intermittent rolling adhesion termed tethering [3]. P-selectin, on the other hand, is primarily found on platelets and endothelial cells where its expression can be constitutive or rapidly upregulated in response to cell activation [1, 5, 6] and facilitates rolling adhesion characterized with intermediate rolling velocities that are typically faster than E-selectin, yet more sustained than tethering adhesion mediated by L-selectin [3].

Under conditions of high shear stress caused by physiological fluid flow, these selectin-mediated interactions are vital to a variety of physiological cell homing processes [7, 8]. For example, endothelial expressed P- and E-selectin are crucial to leukocyte recruitment during inflammation [9, 10], as rolling adhesion facilitated by these molecules precedes firm adhesion and eventual transmigration of circulating cells [11]. In the context of vascular injury, monocyte and platelet accumulation and aggregation at denuded vascular regions also rely on adhesive interactions with P-selectin [12]. While L-selectin expressed on leukocytes can interact with corresponding ligands on the endothelium of high-endothelial venules or the inflamed endothelium of non-lymphoid tissues [13, 14], when expressed by leukocytes adherent to the endothelium, it can additionally facilitate secondary capture of circulating cells [15, 16].

Selectins have also been implicated in the metastatic progression of multiple cancer types, with genetic knockdown or pharmacological inhibition of P-, E-, and L-selectin significantly reducing metastasis to distant organs in *in vivo* metastasis models [17–19]. This is thought to result from direct interactions of metastatic cells with P- and E-selectin expressed on the inflamed vascular endothelium in a manner which facilitates their firm adhesion and eventual transmigration [20, 21]. Indirectly, leukocytes and platelets can enable in a selectin dependent fashion either secondary capture of metastatic cells or the formation of tumor cell emboli to facilitate immune evasion and resist dispersive shear forces in the vasculature [19, 22, 23]. Direct or indirect engagement of metastatic cells with selectins can also confer pro-survival signals to the selectin-engaged tumor cell [24] and can likewise signal to the endothelium for upregulation of chemokines in a manner which promotes a permissive metastatic microenvironment [25].

Accordingly, attenuating selectin-mediated mechanisms of metastatic cell adhesion represents an attractive potential approach for attenuating cancer metastasis and progression. However, a central challenge in the development of selectin-targeting therapeutic strategies remains the potential for deleterious effects of such interventions on normal physiological cell homing. As such, elucidating the manner in which metastatic cell interactions with selectins differ quantitatively and qualitatively in comparison to leukocytic cells has the potential to help inform the development of cell-specific interventions. This pursuit necessitates a platform to interrogate the initiation and sustainment of rolling adhesion mediated by selectins by large numbers of heterogeneous cells per experiment that can be used for the development and dose testing of therapeutics with metastasis-specific inhibition of cell adhesion. To this end, we employed a previously developed cell adhesion chromatography platform and analytical methodology [26] to parse out differences in the efficiency and rolling adhesion qualities of >2×10^4^ metastatic and leukocytic cell subtypes on each P-, E-, and L-selectin. This experimental configuration ensures all assayed cells have uniform contact with a selectin-functionalized substrate to allow direct comparisons in adhesive behavior between assayed cell subtypes. Additionally, the utilization of recombinant protein-functionalized substrates facilitates tight control over the type and density of selectin presentation, and wall shear stress can be easily manipulated by changing the rate of perfusion, parameters that are more difficult if not impossible to manipulate in endothelialized microfluidic devices or *in vivo* experimentation. Using this experimental and analytical technique, we found that diminished rolling adhesion persistence exhibited by metastatic but not leukocytic cell subtypes [26] is most pronounced at low concentrations of P-selectin. In stark contrast to P-selectin, rolling adhesion was found to be highly persistent on E-selectin and reduced on L-selectin, irrespective of cell subtype. Conditions under which adhesion persistence is diminished correspond to those exhibiting the greatest selectin antagonist sensitivity. This data suggests that P-selectin mediated mechanisms of cell homing exhibit the most therapeutically exploitable disparities in metastatic versus leukocytic cell adhesive phenotypes.

## RESULTS

### Leukocytic cells exhibit varied extents of P-, E-, and L-selectin binding in solution, while metastatic cells bind all selectins to similar extents

**Figure 1:**
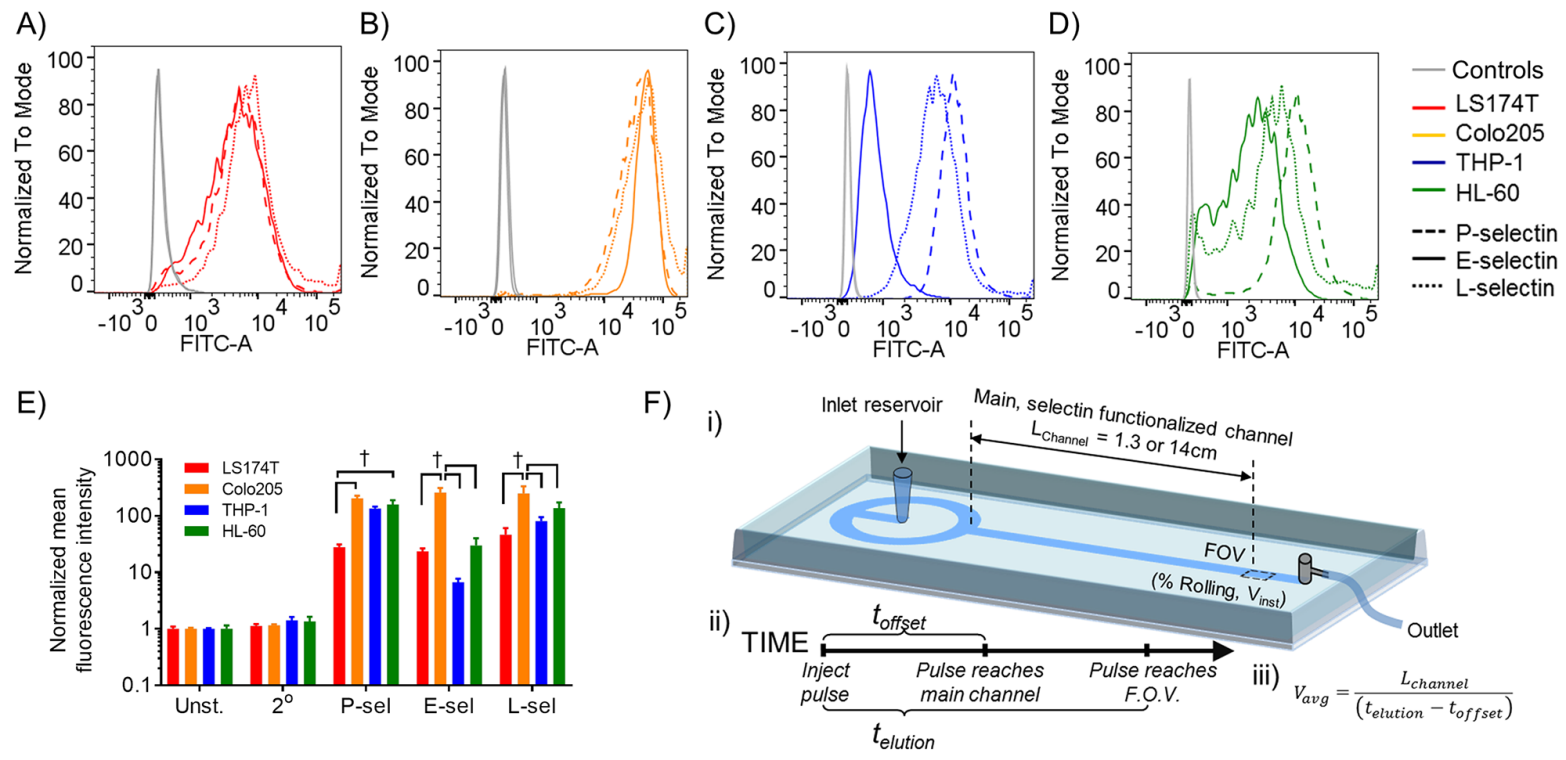
Metastatic and leukocytic cells bind P-, E-, and L-selectin in solution, though to different extents between each cell type **(A-D)** Representative flow cytometry fluorescence intensity distributions for P-, E-, and L-selectin binding in solution, normalized to the mode fluorescence intensity for each group. Controls included both an unstained and secondary-only stained sample. **(E)** Mean fluorescence intensity normalized to unstained sample; two-way ANOVA with Bonferroni correction for multiple comparisons; † indicates significance of comparison between cell subtypes. **(F)** Schematic of experimental setup. (i) A pulse of cell suspension (50 μL of 5×10^5^ cells/mL) is injected into the inlet reservoir and flow is initiated by withdrawing fluid via syringe pump from the outlet. Cells were tracked in the field of view (FOV), and the fraction of cells mediating rolling adhesion (% rolling) as well as individual cell instantaneous velocities (V_inst_) were determined. (iii) Average velocities (V_avg_) were calculated by dividing the length of the functionalized channel (L_channel_) by the difference in elution time (t_elution_) and offset time (t_offset_), each of which are described in the depicted timeline (ii).

In order to begin interrogating cell subtype differences in adhesive interactions with each of the selectins, conventional flow cytometry methods were employed, in which the extent of P-, E-, and L-selectin binding in solution was compared within and between cell types. While metastatic colon carcinoma cell lines (LS174T and Colo205) each exhibited similar extents of P-, E-, and L-selectin binding in solution (Figure 1A–1B), leukocytic THP-1 and HL-60 cells each bound P-selectin to the greatest extent, followed by L-selectin, then E-selectin (Figure 1C–1D). When normalized to unstained and secondary antibody-only controls, Colo205 metastatic cells exhibited significantly higher E- and L-selectin binding ability compared to both THP-1 and HL-60 leukocytic cells (Figure 1E). These data suggest that both metastatic and leukocytic cell subtypes bind P-, E-, and L-selectin, but exhibit cell subtype differences in their ability to bind E- and L-selectin in solution.

### Relationships between rolling adhesion quantities and instantaneous rolling velocities diverge on E-, but not P- or L-, selectin

**Figure 2:**
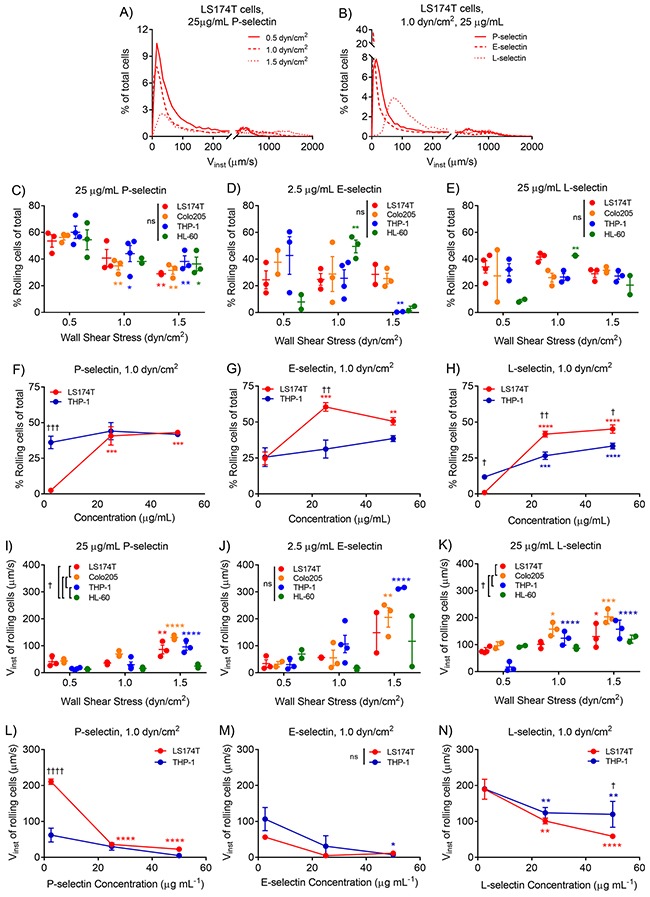
Leukocytic and metastatic cell rolling adhesion quantities and instantaneous velocities are dependent on wall shear stress and selectin concentration Histograms of LS174T cell instantaneous velocities (V_inst_) on substrates functionalized with 25 μg/mL P-selectin at 0.5-1.5 dyn/cm^2^ **(A)** or 25 μg/mL of P-, E-, or L-selectin at 1.0 dyn/cm^2^ **(B)** exhibit two peaks; indicative of a population of cells mediating rolling adhesion (V_inst_<125, 250, 375 at 0.5, 1.0, and 1.5 dyn/cm^2^, respectively) and a population of cells in free flow. At 25 μg/mL P-selectin, increasing wall shear stress reduces the fraction of cells mediating rolling adhesion **(A)**, but does not substantially alter the fraction of cells rolling on either E- **(D)** or L- **(E)** selectin, and there are no differences in the rolling fraction between cell subtypes. At 1.0 dyn/cm^2^, the fraction of metastatic rolling cells increases with increasing selectin concentration for P- **(F)**, E- **(G)**, and L- **(H)** selectin. The instantaneous rolling velocities of both metastatic and leukocytic cell subtypes increase with increasing wall shear stress **(I-K)**, and decrease with increasing selectin concentration **(L-N)**. **(A-B)** Merged data (n ≥ 2 x10^4^ tracked cells) from independently run experiments. **(C-E, I-K)** Data points represent individual, independent experiments, where mean ± SEM is indicated. **(F-H, L-N)** Data represents mean ± SEM. **(C-N)** Two-way ANOVA with Bonferroni correction for multiple comparisons; ^*^ indicates significance of comparison to lowest wall shear stress or concentration, † indicates significance of comparison between cell subtypes either over all wall shear stresses **(C-E, I-K)** or at each concentration **(F-H, L-N)**.

Cell recruitment in the vasculature occurs amidst hemodynamic forces imparted by blood flow, and as such, binding of selectins to cells in solution as in flow cytometry- based assays neglects the effect of wall shear stress on force-dependent selectin-ligand interactions [27–29]. In order to interrogate these interactions in a manner that more accurately recapitulates the hemodynamic environment of the vasculature, we utilized an adhesion chromatography microfluidic device and analytical platform (Figure 1F), developed and described previously [26], in which a pulse of cell suspension is perfused into a non-functionalized settling feature, which ensures uniform cell contact with the substrate prior to entering a main, selectin functionalized channel. The flow rate of perfusion medium or cell suspension through the device was predetermined and set to achieve wall shear stresses ranging from 0.5 to 1.5 dyn/cm^2^, which typify the low fluid forces exhibited in the venous circulation where cell recruitment occurs [18, 27, 28]. Two hour long videos acquired and analyzed using high speed video microscopy and a custom post-processing program revealed a distribution of instantaneous velocities (V_inst_), with a notable proportion of cells exhibiting rolling adhesion, which is characterized by slow, unsteady forward translation at speeds slower than that of cells in free flow (0 < V_inst_ < 125, 250, or 375 μm/sec for 0.5, 1.0, or 1.5 dyn/cm^2^). The bimodal distribution of experimentally measured LS174T metastatic colon carcinoma cell instantaneous velocities in shear flow represents cell populations mediating rolling adhesion versus those in free flow (non-adherent), where the fraction of rolling cells appeared to decrease with increasing wall shears stress (Figure 2A) and roll more slowly on P- and particularly E-selectin than on L-selectin (Figure 2B). The wall shear stress dependency of rolling adhesion quantities of both leukocytic [30] and metastatic cells [31, 32] as well as the distinct rolling adhesion behavior of LS174T cells on each of the selectins, where rolling adhesion is slow and steady on E-selectin, faster on P-selectin, and most sporadic and characteristic of “tethering” on L-selectin have been previously described [3]. Direct, comprehensive comparisons between the rolling adhesion behavior of metastatic and leukocytic cell subtypes on each of the selectins and their dependency on wall shear stress and concentration, however, have not been reported.

Accordingly, we quantified the extent of cell rolling adhesion in shear flow and characterized rolling adhesion velocities of metastatic and leukocytic cell subtypes across wall shear stresses, selectin types, and selectin concentrations. As previously reported, a reduction in the percent of cells mediating rolling adhesion with increasing wall shear stress was observed for all cell subtypes on P-selectin (Figure 2C), but only for THP-1 cells on E-selectin and not for any other cell types on L-selectin (Figure 2D–2E). The shear stress dependency of rolling adhesion quantities on E-selectin was less straightforward (Figure 2D), particularly for HL-60 cells, where rolling efficiency increased from 0.5 to 1.0 dyn/cm^2^, but decreased from 1.0 to 1.5 dyn/cm^2^. Despite these differences in rolling fraction dependency on wall shear stress, the extent of rolling adhesion was similar between metastatic and leukocytic cells at all wall shear stresses, on all selectins (Figure 2C–2E). Increasing the concentration of functionalized selectin increased the proportion of metastatic LS174T rolling cells on all of the selectins, but only increased the fraction of rolling leukocytic THP-1 cells on L-selectin (Figure 2F–2H). Within this rolling population, instantaneous velocities of both cell subtypes on all selectins were directly proportional to wall shear stress, which is in agreement with published reports [7, 33], but metastatic Colo205 cells exhibited higher velocities relative to both THP-1 and HL-60 leukocytic cells on P- and L-selectin, but not E-selectin (Figure 2I–2K). Furthermore, cells exhibited reductions in instantaneous rolling velocities with increasing selectin concentrations, particularly on P- and L-selectin (Figure 2L–2N). Taken together, these data suggest that cell subtype differences in rolling instantaneous velocities, but not necessarily rolling adhesion quantities emerge mostly on P- and L-selectin.

**Figure 3:**
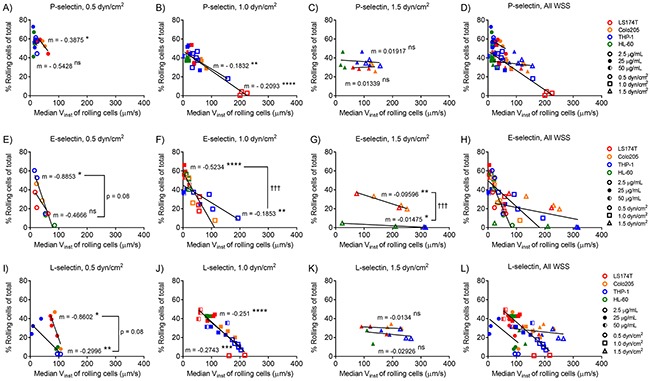
Divergence of relationships between rolling adhesion quantities and instantaneous rolling velocities with cell subtypes varies on P-, L-, and E-selectin and with wall shear stress The median instantaneous velocity of cells mediating rolling adhesion is inversely proportional to the percentage of rolling cells of total on P- **(A-D)**, E- **(E-H)**, and L- **(I-L)** selectin, and the slopes of these relationships decrease with increasing wall shear stress. **(A-C, E-G, I-K)** Linear regression with slopes reported separately for grouped metastatic (LS174T and Colo205) and leukocytic (THP-1 and HL-60) cells, significantly non-zero slopes indicated with a ^*^, and significant comparisons between the slopes of regressions for cell subtypes indicated with a †. **(A-L)** Data points represent individual, independently run experiments.

Given these modest differences in cell subtype adhesive behavior on P-selectin, we sought to determine if the relationship between instantaneous velocity and percentage of rolling cells similarly diverged by cell type. Holistically, we found these quantities to be inversely proportional (Figure 3), with slopes that decrease with increasing wall shear stress (Table 1). However, while instantaneous velocity diverged between cell subtypes at low concentrations of P-selectin, the relationship between instantaneous velocity and the percent of cells mediating rolling adhesion diverged only on E-selectin and to a lesser extent at low wall shear stresses on L-selectin (Figure 3E–3G and 3I). These results suggest that metastatic and leukocytic cells exhibit selectin-dependent differences in the extent and velocity of rolling adhesion.

**Table 1:** Slopes of mean cell instantaneous velocity versus percent rolling decrease with increasing wall shear stress

Selectin	τ_wall_ (dyn/cm^2^)	Slope	r	Sig.
P	0.5	-0.17	-0.32	ns
1	-0.21	-0.91	^****^	
1.5	-0.034	-0.24	ns	
E	0.5	-0.49	-0.47	ns
1	-0.27	-0.79	^****^	
1.5	-0.036	-0.29	ns	
L	0.5	-0.23	-0.48	ns
1	-0.25	-0.92	^****^	
1.5	-0.034	-0.29	ns	

Summary of slopes from linear regressions and Pearson's r values for relationships between instantaneous velocity and percent rolling cells shown in Figure 3D, 3H, and 3L for all cell subtypes pooled and grouped by wall shear stress. ^*^ Indicates significance of Pearson's r correlation.

### Average and instantaneous velocities diverge on P-selectin for metastatic cells and L-selectin for metastatic and leukocytic cells

**Figure 4:**
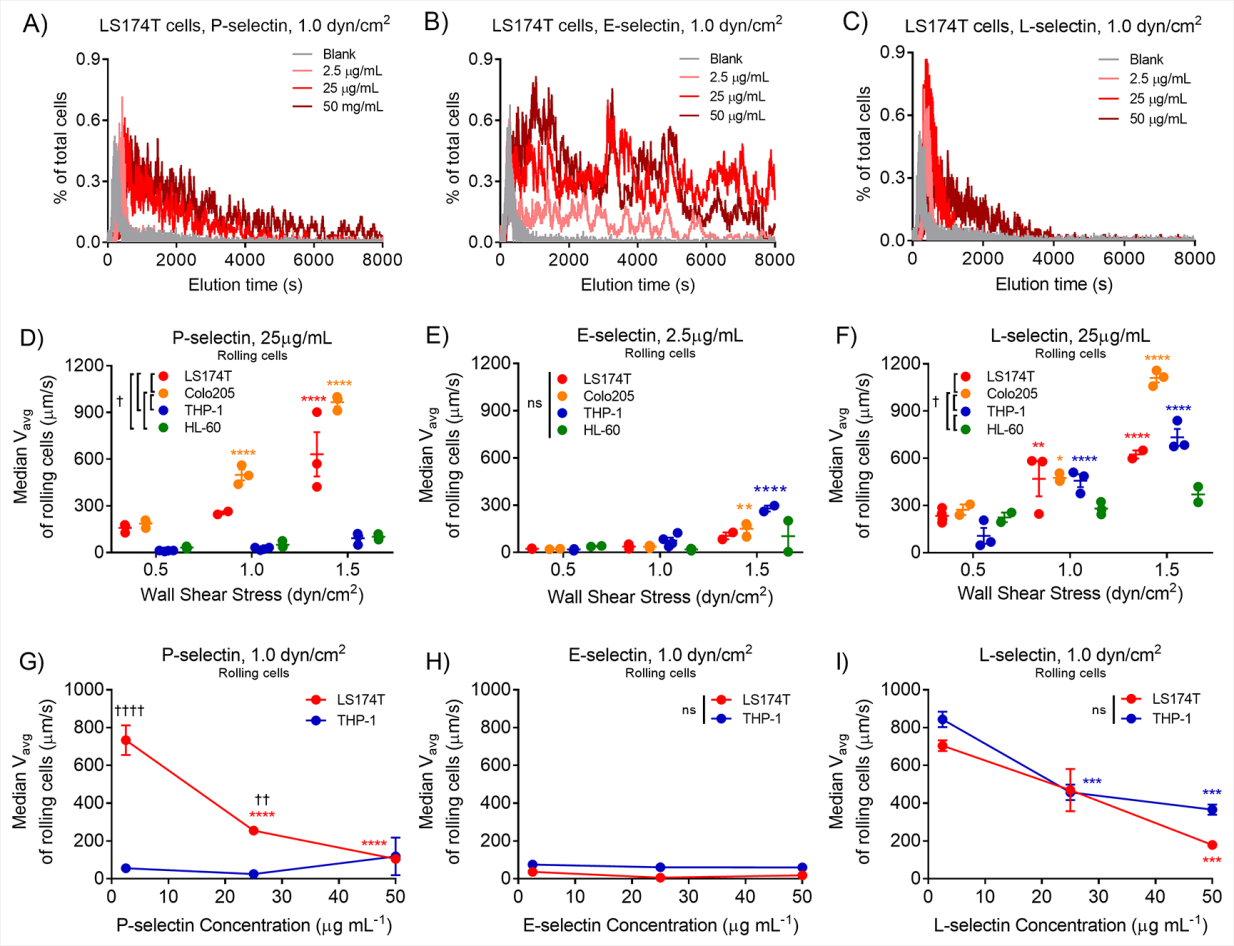
Differences in metastatic versus leukocytic cell average velocities are greatest on low concentrations of P-selectin at high wall shear stresses **(A-C)** Representative residence time distributions for LS174T cells on 2.5, 25, and 50 μg/mL P-, E-, and L-selectin. Increases in wall shear stress result in increased average velocities of metastatic cell subtypes on P- selectin **(D)**, marginal increases on E-selectin **(E)**, and increased average velocity of both cell subtypes on L-selectin **(F)**. Likewise, increasing P-selectin concentration reduces average velocities of metastatic, but not leukocytic cell subtypes on P-selectin **(G)**, neither cell subtype on E-selectin **(H)**, and both cell subtypes on L-selectin **(I)**. **(A-C)** Representative data. **(D-F)** Data points represent individual, independently run experiments, where mean ± SEM is indicated. **(G-I)** Data represents mean ± SEM. **(D-I)** Two-way ANOVA with Bonferroni correction for multiple comparisons; ^*^ indicates significance of comparison to lowest wall shear stress or concentration, † indicates significance of comparison between cell subtypes either over all wall shear stresses **(D-F)** or at each concentration **(G-I)**.

In order to gain insight into the average, rather than instantaneous, adhesive behavior of each cell subtype on P-, E-, and L-selectin over the entire channel length versus imaging field of view, we next analyzed the elution time behavior of cells (e.g. arrival within the field of view at the end of the selectin-functionalized channel). As expected, LS174T cells perfused through non-functionalized channels (blank), exhibited residence time distribution profiles that showed the entire cell population eluting within the first few minutes of perfusion, whereas increasing P-, L-, and to a lesser extent E-selectin concentrations facilitated adhesion in a manner which increased the residence time of cells in the field of view (Figure 4A–4C). Elution times were utilized to determine average velocities on a single cell basis (Equation 1, Methods), which were found to be significantly higher for both LS174T and Colo205 metastatic colon carcinoma cells relative to THP-1 or HL-60 leukocytic cells at 25 μg/mL P-selectin and increased with increasing wall shear stress (Figure 4D). 25 μg/mL L-selectin facilitated similar average velocity increases with shear stress for all but HL-60 cells, and metastatic Colo205 cells exhibited higher average velocities relative to either leukocytic cell subtype (Figure 4F). However, there were no cell subtype differences in average velocity on 2.5 μg/mL E-selectin, and only modest shear stress dependence (Figure 4E). E- and L-selectin concentrations did not affect average velocities of either cell subtype, whereas cell subtype differences in average velocity were exaggerated at low concentrations of P-selectin (Figure 4G–4E).

**Figure 5:**
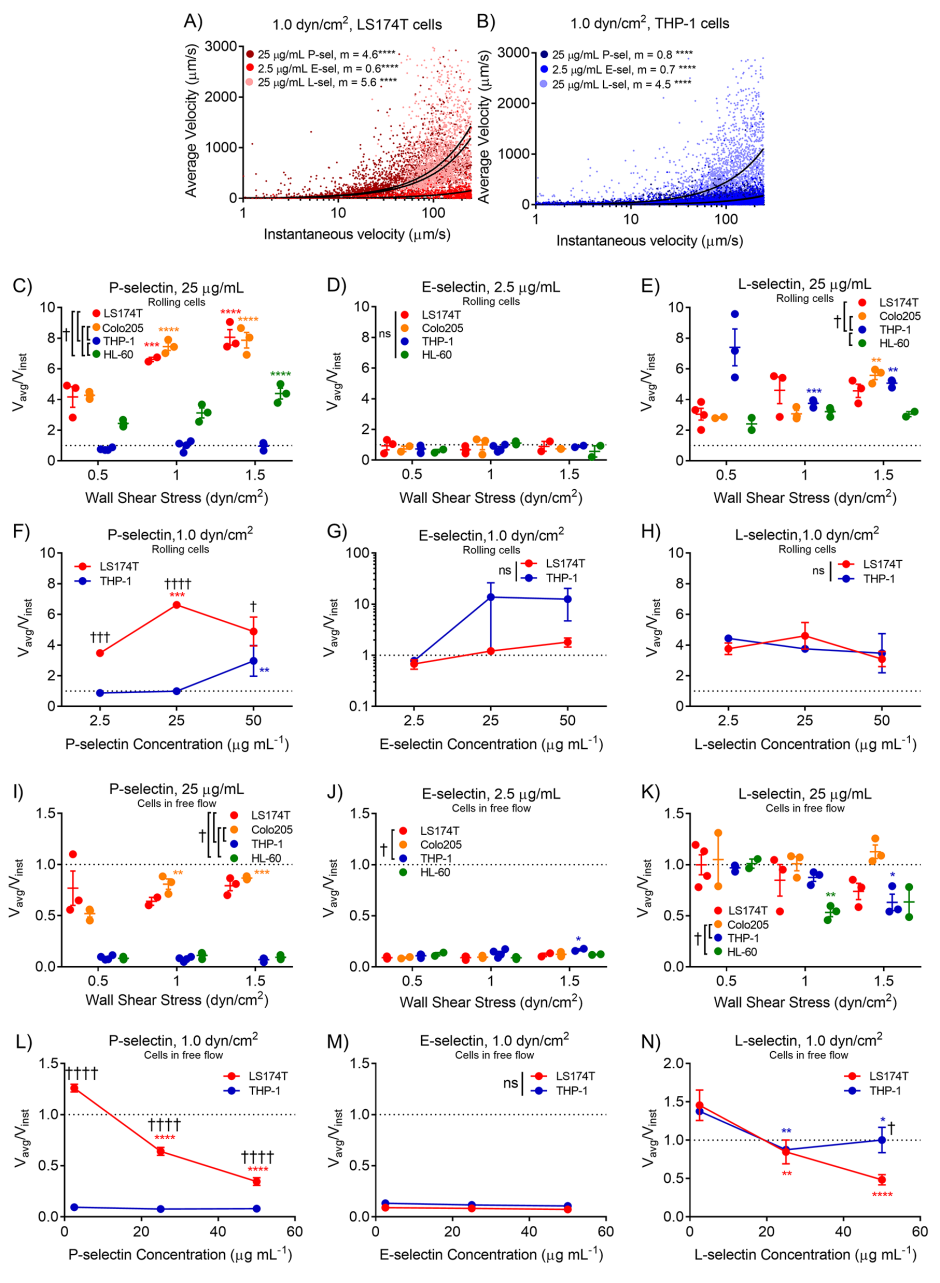
Average velocities diverge from measured instantaneous velocities of metastatic cells on P-selectin and both metastatic and leukocytic cells on L-selectin in rolling adhesion **(A-B)** The average velocities computed from elution times increase linearly with instantaneous velocity, where the slope of this relationship varies by cell and selectin type. The ratio of average to instantaneous velocities of rolling cells increases with increasing wall shear stress for metastatic cells and is higher than that of leukocytic cells on P-selectin **(C)**, but not E- **(D)** or L- **(E)** selectin. P- **(F)** E- **(G)**, and L- **(H)** selectin concentration have little effect on this ratio. The average velocity of metastatic cells in free flow more accurately predicts their instantaneous velocity on P-selectin at all wall shear stresses. **(I)** These ratios for cells in free flow are less than one for both cell subtypes on E-selectin and show minimal shear stress or cell subtype dependency **(J)**, but are close to one for both cell subtypes on L-selectin **(K)** and exhibit negligible wall shear stress dependence. Increasing selectin concentration reduces the V_avg_/V_inst_ ratio of metastatic cells in free flow on P-selectin **(L)** and both metastatic and leukocytic cells on L-selectin **(N)**, while E-selectin concentration exhibits negligible effects **(M)**. **(A-B)** Representative data; linear regression, ^*^ indicates non-zero slope. **(C-N)**. Dotted lines indicate V_avg_/V_inst_ of 1. **(C-E, I-K)** Data points represent individual, independently run experiments, where mean ± SEM is indicated. **(F-H, L-N)** Data represents mean ± SEM. **(C-N)** Two-way ANOVA with Bonferroni correction for multiple comparisons; ^*^ indicates significance of comparison to lowest wall shear stress or concentration, † indicates significance of comparison between cell subtypes either over all wall shear stresses **(C-E, I-K)** or at each concentration **(F-H, L-N)**.

Interestingly, a comparison of computed average velocities with measured instantaneous velocities on a single cell basis revealed that while higher average velocities generally corresponded to higher instantaneous velocities, the slopes of these relationships indicated that the average velocity does not directly predict the instantaneous velocity for all cell subtypes and selectins (Figure 5A–5B). More specifically, the average velocity of metastatic LS174T and Colo205, but not leukocytic THP-1 cells, on 25 μg/mL P-selectin overestimated their instantaneous velocity (V_avg_/V_inst_>1, Figure 5C), while on 2.5 μg/mL E-selectin, the average and instantaneous velocities of all cell subtypes were approximately equal, and their ratio did not depend on wall shear stress (Figure 5D). On 25 μg/mL L-selectin, V_avg_/V_inst_ of rolling cells indicated that average velocity overestimated instantaneous velocity of all cell subtypes, particularly at higher wall shear stresses (Figure 5E). While concentration had minimal effect on this ratio for either cell type on E- or L-selectin, differences in V_avg_/V_inst_ between cell subtypes on P-selectin were most pronounced at low selectin concentrations (Figure 5F–5H). When considering the population of cells in free flow, the average velocity of metastatic cells much more closely approximated their instantaneous velocity at all wall shear stresses on P-selectin (Figure 5I), neither cell subtype on E-selectin (Figure 5J), and both cell subtypes on L-selectin (Figure 5K). The V_avg_/V_inst_ ratio of cells in free flow exhibited selectin concentration dependence on both P- and L-selectin (Figure 5L–5N). These data suggest that neither instantaneous nor average velocity accurately describes all cell adhesive behavior over long lengths. As described previously [26], a cell may switch between rolling adhesion and free flow states many times over the length of a channel, such that a cell may not arrive in the field of view at a time predicted by its instantaneous velocity, thus contributing to a mismatch between average and instantaneous velocities.

### Mean percent binding time is reduced for metastatic cells on P-selectin and both metastatic and leukocytic cells on L-selectin

**Figure 6:**
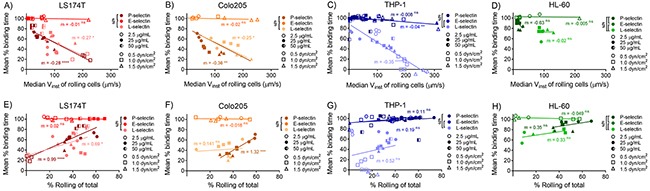
Relationships between mean percent binding time and instantaneous velocity or rolling efficiencies diverge between selectins for each cell subtype Mean percent binding time is proportional to the median instantaneous velocity of rolling cells **(A-D)** and percent rolling cells of total **(E-H)** for all cell subtypes. Relationships between mean percent binding time and either instantaneous rolling velocity or percent rolling are most similar for metastatic cells on P- and L-selectin **(A-B, E-F)** and for leukocytic cells on P- and E-selectin **(C-D, G-H)**. **(A-H)** Each point represents an individual, independently run experiment. Linear regression, ^*^ indicates non-zero slope, § indicates significance of comparison between selectins by one-way ANOVA with Bonferroni correction for multiple comparisons.

In order to more comprehensively describe cell adhesive behavior over longer lengths for more direct comparisons of adhesive behavior between cell subtypes, we employed a previously developed metric, mean percent binding time, which estimates the fraction of time a cell spends engaged in adhesive interactions with the selectin-functionalized substrate [26]. For both LS174T and Colo205 metastatic cells, mean percent binding time similarly decreased with increasing instantaneous velocity and increased with increasing fraction of rolling cells on both P- and L-selectin, but not E-selectin (Figure 6A–6BA, 6E–6FE). However, leukocytic THP-1 cells exhibit mean percent binding times that only minimally change with either instantaneous velocity or the percentage of rolling cells on P- or E-selectin, but more substantially with L-selectin (Figure 6C and 6G). HL-60 leukocytes exhibit trends similar to those of THP-1 cells, though their slopes are not statistically non-zero (Figure 6D and 6H). These data suggest that high mean percent binding time is facilitated when a large proportion of cells are rolling at slower velocities.

**Figure 7:**
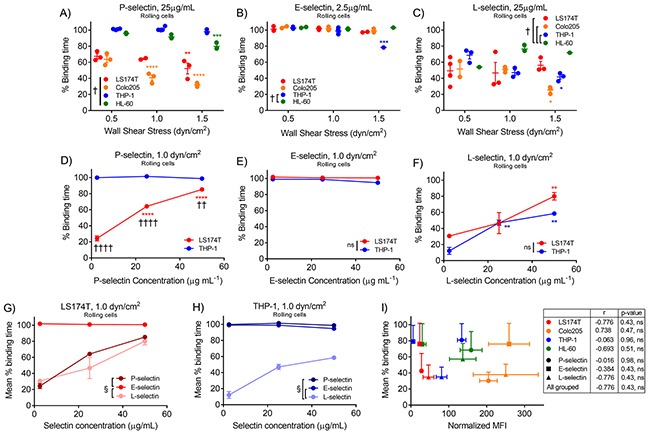
Metastatic cells exhibit reduced rolling adhesion persistence on P- and L-selectin, but not E-selectin, in a manner which is dependent on selectin concentration **(A)** The mean percent binding time is reduced for metastatic relative to leukocytic cells on P-selectin, particularly at high wall shear stresses. **(B)** On E-selectin, both cell subtypes exhibit high mean percent binding time with negligible shear stress dependence. **(C)** Both metastatic and leukocytic cells exhibit reduced percent binding time on L-selectin. Increased concentrations of P-selectin restore metastatic cell persistence to levels similar to that of THP-1 cells **(D)**, while increased concentrations of E-selectin have no affect on mean percent binding time **(E)**. **(F)** Increases in L-selectin concentration yield increased persistence of both cell subtypes. The relationship between selectin concentration and mean percent binding time is most similar on P- and L-selectin for metastatic cells **(G)**, but P- and E-selectin for non-metastatic cells **(H)**. **(I)** The mean percent binding time at 1.0 dyn/cm^2^ and 25 μg/mL P-selectin, 2.5 μg/mL E-selectin, or 25 μg/mL L-selectin does not correlate with mean fluorescence intensity (MFI) measured via flow cytometry. **(A-C)** Data points represent individual, independently run experiments and mean ± SEM is indicated. **(D-I)** Data represents mean ± SEM. **(A-G)** Two-way ANOVA with Bonferroni correction for multiple comparisons; ^*^ indicates significance of comparison to lowest wall shear stress or concentration, † indicates significance of comparison between cell subtypes or selectins either over all wall shear stresses **(A-C, G-H)** or at each concentration **(D-F)**. **(I)** Pearson's correlation indicated by grouping.

Mean percent binding time of each cell subtype was next evaluated over a range of wall shear stresses, selectin types, and selectin concentrations. As we previously reported [26], mean percent binding time on P-selectin was reduced for metastatic cell subtypes, in a manner most exaggerated at higher wall shear stresses and lower P-selectin concentrations (Figure 7A and 7D). On E-selectin, all cell subtypes exhibited approximately 100% binding time at nearly all wall shear stresses and E-selectin concentrations (Figure 7B and 7E), whereas L-selectin facilitates cell rolling adhesion with reduced persistence, particularly at low selectin concentrations, with only subtle differences between cell subtypes (Figure 7C and 7F). The relationship between increasing selectin concentration and increased mean percent binding time is similar for LS174T cells on P- and L-selectin and for THP-1 cells on P- and E-selectin, revealing a divergence in the way metastatic versus leukocytic cells interact with the selectins holistically (Figure 7G and 7H). The percent binding time of cells in shear flow with selectin-functionalized surfaces did not correlate with binding to selectins in solution assayed by flow cytometry (Figure 7I), nor did the percentage of cells rolling, instantaneous velocity, or average velocity (data not shown).

### Heparin attenuates rolling adhesion persistence of metastatic cells on P-selectin and both metastatic and leukocytic cells on L-selectin

**Figure 8:**
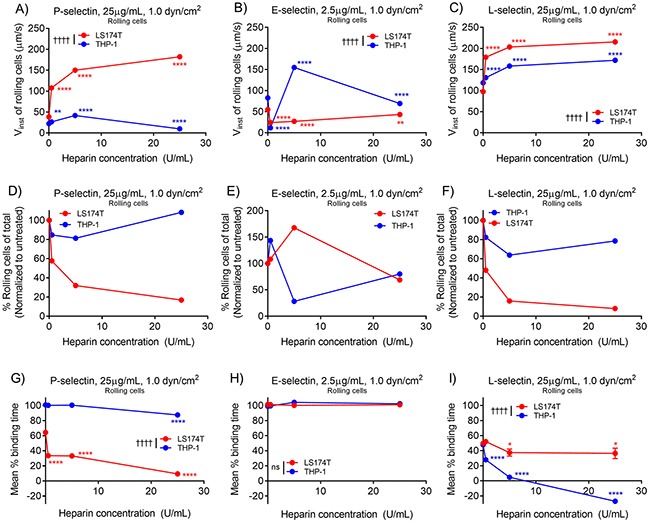
Heparin increases metastatic cell rolling adhesion velocity, diminishes rolling adhesion quantities, and reduces rolling adhesion persistence on P- and L-selectin, but not E-selectin The median instantaneous rolling velocity of metastatic cells on P-selectin **(A)** and both metastatic and leukocytic cells on L-selectin **(C)** increases with increasing heparin concentration, while instantaneous velocities of both cell subtypes on E-selectin do not linearly depend on heparin concentration **(B)**. The fraction of rolling cells and mean percent binding time of metastatic, but not leukocytic cells decreases with increasing heparin dose on P-selectin **(D, G)** though highest tested concentrations of heparin marginally reduced persistence of leukocytic cells. On E-selectin, increasing heparin dose did not alter the fraction of rolling cells in a correlative fashion **(E)**, nor did it reduce percent binding time **(H)** of either LS174T metastatic or THP-1 leukocytic cells. On L-selectin, both the fraction of rolling cells **(F)** and mean percent binding time **(I)** decrease with increasing heparin dose for both cell subtypes. **(A-I)** Data represent mean ± SEM. Two-way ANOVA with Bonferroni correction for multiple comparisons; ^*^ indicates significance of comparison to lowest concentration, † indicates significance of comparison between cell subtypes over all heparin doses.

We next evaluated the effect of low-dose heparin, a known P- and L-selectin antagonist [19, 34], on the capacity of metastatic versus leukocytic cells to mediate and sustain rolling adhesion with P-, E-, and L-selectin. On P-selectin, we found that metastatic cell instantaneous velocity was increased, rolling fraction decreased, and mean percent binding time reduced with increasing doses of heparin, while leukocytic THP-1 mean percent binding time was only marginally diminished at maximum doses of heparin evaluated (Figure 8A, 8D, and 8G). Instantaneous velocities and rolling percentages of both cell subtypes on E-selectin did not exhibit clear effects of heparin treatment, but mean percent binding time clearly indicated that the rolling adhesion persistence of both cell subtypes on E-selectin was not affected by heparin treatment (Figure 8B, 8E, and 8H). On L-selectin, both metastatic LS174T and leukocytic THP-1 cell instantaneous velocities were increased, rolling percentages decreased, and mean percent binding time reduced with increasing heparin dose (Figure 8C, 8F, and 8I). Taken with earlier data demonstrating reduced mean percent binding time of metastatic cells on P-selectin and both metastatic and leukocytic cells on L-selectin, these findings suggest that heparin imparts the greatest influence on rolling adhesion behavior of cell subtype-selectin pairs that already exhibit reductions in rolling adhesion persistence in the absence of heparin.

## DISCUSSION

In this work we utilized a previously developed cell adhesion chromatography platform and analytical method [26] to analyze cell subtype differences in the efficiency and sustainment of rolling adhesion on P-, E-, and L-selectin. We found that frequencies of rolling adhesion were inversely proportional to wall shear stress for all cell subtypes on P-selectin and for THP-1 cells on E-selectin but did not exhibit linear dependence for the remaining cell subtypes on E- or L-selectin. Both measured instantaneous velocities and estimated average velocities of all cell subtypes generally increased with increasing wall shear stress, decreased with selectin concentration, and differed between cell subtypes on P- and to a lesser extent L-selectin (Figures 2 and 4). Quantification of the mean percent of time of cell engagement with the selectin-functionalized substrate in flow revealed that only on P-selectin, particularly at lower concentrations, but not on E- or L-selectin, did disparities in the adhesion persistence of metastatic versus leukocytic cell subtypes emerge (Figures 6–7).

By raising the probability of forming the minimum number of bonds required to resist the dispersive forces of fluid flow, lower wall shear stress and higher surface presented concentrations of selectin have been shown to increase cell rolling adhesion frequencies [30]. In agreement with this, we observed a reduction in the percent of cells mediating rolling adhesion with increasing wall shear stress for all cell subtypes on P-selectin and THP-1 cells on E-selectin (Figure 2C–2D). However, rolling frequencies of the other assayed cell types exhibited less straightforward relationships with shear stress (Figure 2D–2E). These may arise as a result of firm adhesion by cells upstream of the imaging FOV in the experimental setup used herein, thereby lowering the overall level of measured rolling adhesion, despite higher overall levels of total adhesion. However, this is most likely only in the case of leukocytic cells, given their higher propensity to mediate firm adhesion and very slow rolling adhesion on selectins. Alternatively, fast kinetic on-rates of selectin-ligand pairs may overcome any increased dispersive fluid forces imparted by increases in wall shear stress over the range interrogated herein [35].

Characterization and modeling of selectin-ligand bond kinetics and mechanics have provided context for the divergence of rolling adhesion behavior of leukocytes on P-, E-, and L-selectin [36, 37] and likewise offer an opportunity to contextualize differences in the rolling adhesion behavior of metastatic cells. For example, our findings corroborate experimentally verified models describing a reduction in the percentage of cells mediating rolling adhesion with increasing instantaneous velocities (Figure 3) [36, 38]. These models have also suggested that faster rolling adhesion is facilitated by higher kinetic off rates and higher reactive compliance, while the frequency of cell rolling adhesion is determined by receptor and ligand densities and the shear rate [38]. Thus, disparities in any of these parameters may affect the slope of the relationship between percent rolling and instantaneous velocity. Indeed, our findings revealed a divergence in the slopes of these relationships for metastatic versus leukocytic cells on E-selectin (Figure 3E–3G), which may be attributed to discrepancies in off-rates reported for E-selectin with Colo205 and HL-60 cells (0.44 and 0.92 s^-1^, respectively) [39] as well as the somewhat diminished E-selectin ligand density on leukocytic versus metastatic cells revealed by flow cytometry staining (Figure 1E). However, slopes for metastatic and leukocytic cells interacting with P-selectin do not significantly differ, despite even greater disparities in the off-rates of P-selectin-leukocyte and P-selectin-LS174T interactions (0.20 and 2.78 s^-1^, respectively) [40]. It is unclear why these differences in off-rates or differences in P-selectin-leukocyte versus -LS174T bond strength (approximately 150 versus 80 pN, respectively at a loading rate of 1000 pN/s) [40, 41] do not produce different slopes of the relationship between instantaneous velocity and rolling frequencies, although smaller differences in measured ligand densities for P- and L- relative to E-selectin between cell subtypes (Figure 1E) and differences in the density of bonds formed during rolling adhesion may functionally contribute.

Receptor-ligand kinetics and mechanics are not only relevant to the propensity to initiate rolling adhesion and control instantaneous rolling velocity, but also may contextualize differences in the ability of metastatic versus leukocytic cells to sustain rolling adhesion. For example, observations of reduced persistence of leukocytic cells on L-, but not P- or E-, selectin (Figure 7) may correspond to the lower tensile strength of leukocyte interactions with L-selectin relative to either P- or E-selectin (approximately 90 versus 140 and 150 pN, respectively at a loading rate of 1000 pN/s) [36, 42] or the inability of L-, but not P- or E-, selectin to form dimerized bonds with leukocytic cell-expressed P-selectin glycoprotein ligand-1 [43–46]. The lower tensile strength reported for P-selectin-LS174T versus P-selectin-PMN interactions (approximately 80 versus 150 pN, respectively at a loading rate of 1000 pN/s) [40, 41] similarly corresponds to our findings of reduced rolling adhesion persistence of metastatic but not leukocytic cells on P-selectin. However, despite characterization of bond mechanics of some metastatic cell selectin ligands such as podoxycalin-like protein (PODXL) with E- and L-selectin [47, 48], the rupture force and dimerization potential of other metastatic cell selectin ligands such as CD44 and carcinoembryonic antigen (CEA) [3, 33, 47] with each E- and L-selectin remains to be determined. Importantly, other cell subtype differences such as cell size [26], deformability [49], and ability to extend cellular projections of clustered ligands [50–52] may synergistically contribute to the observed rolling adhesion behavior. As such, though precise underpinnings of adhesion persistence remain unclear, the inability of in-solution flow cytometry assays to accurately predict adhesion persistence (Figure 7I) underscores the value of assaying cell-selectin interactions in a hemodynamically relevant context.

While the results reported throughout this work focus on human colon carcinoma cell lines due to the highly metastatic nature of this cancer type [53] and implicated role of selectins in its hematogenous metastasis [54], the rolling adhesion persistence of other metastatic cell subtypes may follow similar trends. For example, breast cancer cell lines reportedly lack PSGL-1 [55], but express CD44 [56], CEA [57], and PODXL [58], much like LS174T cells [3, 33, 47], which may imply that their rolling adhesion behavior exhibits similar trends on P-, E- and L-selectin. However, the extent to which the type and density of selectin ligand expression versus other cell characteristics regulate a cells ability to initiate and sustain rolling adhesion remains unclear. Accordingly, the approach described in this work may offer standardized metrics by which a more holistic description of rolling adhesion behavior of other metastatic cell subtypes can be characterized.

The experimental and analytical technique presented herein furthermore enabled for a direct comparison of the effects of heparin on metastatic versus leukocytic cell adhesion to each of the selectins. Given its ability to reduce metastatic spread in *in vivo* models by inhibiting L-selectin mediated adhesion and interfering with P-selectin mediated aggregation of platelets on tumor cells [19, 59], heparin has been explored for its potential as an anti-metastatic therapeutic. However, heparin has also been shown to attenuate P- and L-selectin mediated adhesion of leukocytic cells [34, 60–63], the recruitment of which is indispensable to the maintenance of homeostasis [8, 10, 14] and furthermore has been implicated in preventing the progression of metastasis by engulfing tumor debris and recruiting other cytotoxic cell subtypes [64]. Accordingly, understanding the efficacy and dose-effects of heparin or other anti-metastatic drug candidates on selectin-mediated interactions of metastatic versus leukocytic cells represents an important step in the development of successful anti-metastatic therapeutics. Our findings revealed that low dose heparin treatment diminished metastatic cell rolling adhesion persistence on both P- and L-selectin, while heparin treatment mainly compromised leukocytic rolling adhesion on L-selectin (Figure 8). Since targeted selectin knockout models have suggested that P-selectin plays a more indispensable role in leukocyte recruitment than L-selectin [10, 65], despite general effects on rolling adhesion persistence on L-selectin, selective attenuation of metastatic rolling adhesion on P- selectin suggests that heparin may selectively attenuate rolling adhesion persistence of metastatic cells on both P- and L- selectin, and would have a less detrimental effect on perhaps functionally redundant L-selectin-mediated interactions by leukocytic cells.

Our results revealed a striking similarity in the relationships of mean percent binding time of LS174T and Colo205 metastatic cells on P-selectin with instantaneous velocity, rolling percentages, and selectin concentration sensitivity with these same relationships for L-selectin-mediated adhesion (Figures 6A–6H and 7G–7HG). Since L-selectin is primarily responsible for tethering and secondary cell capture of leukocytes at later time points in the cell recruitment processes [15, 65, 66] and can mediate adhesion with leukocytes in free flow [67], the similarity of metastatic cell adhesion persistence facilitated by P-selectin to that of L-selectin may suggest that P-selectin has a higher potential to functionally facilitate heterotypic aggregation of metastatic cells with activated platelets via platelet expressed P-selectin rather than mediating direct, sustained rolling adhesion on the inflamed endothelium. Indeed, McCarty et al. reported that metastatic LS174T and Colo205 cells initially tether to P-selectin expressed by surface-immobilized platelets in a manner that is stabilized by engagement of von Willebrand factor [31].

In conclusion, using a cell adhesion chromatography experimental and analytical platform to assay large quantities of cells comprising heterogeneous cell populations for comparison of their ability to engage in and sustain rolling adhesion on P-, E-, and L-selectin over a range of wall shear stresses and selectin concentrations, this study revealed a divergence in the rolling adhesion persistence of metastatic versus leukocytic cells on P-selectin, but not E- or L-selectin. While this disparity was most exaggerated at low P-selectin concentrations, both cell subtypes exhibited high, nearly 100% persistence on E-selectin and reduced persistence on L-selectin, regardless of selectin concentration, an effect that could not be predicted by flow cytometry analysis of cell surface-expressed selectin ligand levels. Reduced persistence corresponded to the greater sensitivity to selectin antagonism with heparin, thereby implicating P-selectin mediated adhesion mechanisms as a therapeutically exploitable target. Overall, our results implicate the utility of the analytical technique presented herein, in which the ability of different cell subtypes to initiate and sustain rolling adhesion on P-, E-, and L-selectin is assayed in a manner that allows for standardized comparisons of mean percent binding time in defining functional differences in cell adhesive phenotypes, particularly in the context of screening the cell-specificity of selectin antagonizing therapeutics.

## METHODS

### Reagents and materials

Cell culture reagents were purchased from Life Technologies (Carlsbad, CA). Cell lines were obtained from the American Type Culture Collection (Manassas, VA). Anti-Human IgG (Fc specific) and Bovine Serum Albumin (BSA) were from Sigma-Aldrich. P-selectin-IgG Fc (P-selectin), E-selectin-IgG Fc (E-selectin), and L-selectin-IgG Fc (L-selectin) were purchased from R&D Systems (Minneapolis, MN). Polydimethylsiloxane (PDMS) base and curing agent were from Ellsworth Adhesives (Germantown, WI). Non-tissue culture treated polystyrene plates (245 mm x 245 mm) were from Corning (Corning, NY).

### Cell culture

Human monocytic THP-1 and human promyeloblast HL-60 cells were cultured in suspension in RPMI1640 supplemented with 10% heat-inactivated fetal bovine serum, 1mM sodium pyruvate, 10 mM 4-(2-hydroxyethyl)-1-piperazineethanesulfonic acid (HEPES), and 1% penicillin-streptomycin. THP-1 and HL-60 cells were subcultured every third day via 1:5 dilution in order that they be maintained between 2 × 10^5^ and 2 × 10^6^ cells/mL. Human colorectal adenocarcinoma LS174T cells were cultured in Dulbecco's Modified Eagle Medium supplemented with 10% heat-inactivated fetal bovine serum (FBS) and 1% antibiotic-antimycotic and Colo205 cells were cultured in RPMI 1640 supplemented with 10% heat-inactivated FBS and 1% antibiotic-antimycotic. LS174T cells were harvested via mild trypsinization (0.25% Trypsin/EDTA at 37°C), centrifuged at 300 x g for 5 minutes and resuspended in complete medium for subculture or resuspended and maintained at 37°C for two hours with continuous resuspension every 15 minutes to allow for regeneration of surface glycoproteins for use in experiments. For Colo205 cells, the suspension cell fraction was collected prior to mild trypsinization of adherent cells. Suspension and adherent fractions were centrifuged together at 300 x g for 5 minutes and similarly resuspended in complete medium for subculture or regeneration of surface glycoproteins. Just prior to experiments, all cells were centrifuged at 300 x g for 5 minutes and resuspended in 0.1% BSA in D-PBS containing calcium and magnesium at 5×10^5^ cells/mL.

### Flow cytometry

P-, E-, and L-selectin ligand expression were measured via flow cytometry. 20 μg/mL P-, E-, and L-selectin Fc chimeras were premixed 1:1 with FITC-anti IgG (Fc specific) in 0.1% BSA in D-PBS with calcium and magnesium for 1 hour at room temperature. LS174T, Colo205, THP-1, and HL-60 cells were resuspended in selectin-IgG premix solutions at 2.5 x10^6^ cells/mL for 1 hour on ice. Cells were subsequently washed and resuspended in D-PBS for analysis on the BD LSRFortessa (BD Biosciences, San Jose, CA, USA).

### Chamber fabrication and functionalization

A custom designed chamber consisting of a 2 mm wide, 100 μm deep, 10.9 mm long linear inlet region followed by a bifurcation into a circular settling feature with inner and outer radii of 10.5 and 11.75 mm, respectively, upstream of a 2 mm wide, 100 μm deep main channel of either 1.3 or 14 cm in length was used [26]. PDMS base and curing agent were mixed at a ratio of 9:1, poured into aluminum channel molds, and cured at 90°C for three hours. Inlet and outlet holes were created with a biopsy punch and channels were bonded to polystyrene plates by spin coating (WS-400BZ-6NPP-LITE, Laurell, North Wales, PA) a 10:1 ratio of PDMS base to curing agent on glass slides, stamping the cured PDMS block in uncured PDMS (functioning as glue), and carefully placing on the polystyrene plate, ensuring no air bubbles formed between the plate and the PDMS block. Dishes were placed in a 50°C oven overnight to allow PDMS glue mixture to cure. Channels were allowed to cool and then were either used immediately or functionalized. For functionalization, the main channel of the chamber was incubated overnight at 4°C with anti-IgG (Fc specific) in D-PBS without calcium and magnesium at concentrations corresponding to the desired selectin concentration. The main channel was subsequently washed with D-PBS, blocked with 1% BSA in D-PBS for 1 hour at room temperature, washed with D-PBS again, incubated with either 2.5, 25, or 50 μg/mL of P-, E-, or L-selectin in D-PBS with calcium and magnesium for 2 hours at room temperature, and washed again with D-PBS. Finally, the entire device, including the settling feature, was blocked with 1% BSA in D-PBS for 1 hour at room temperature, washed with D-PBS, and stored at room temperature until use in same-day experiments.

### Perfusion experiments

An inlet reservoir was installed upstream of the chamber's settling feature and an outlet tubing line was filled with 0.1% BSA in D-PBS perfusion medium and connected to the outlet of the D-PBS-filled chamber, taking care to ensure no air bubbles formed at connections. The outlet line was connected to a syringe on a PhD Ultra Harvard Apparatus syringe pump (Holliston, MA) via Luer-lock connection, and the chamber was placed on an optical microscope (Eclipse Ti, Nikon, Melville, NY). The focal plane was determined in advance by focusing on the bottom of the chamber and raising it 5 μm while 0.1% BSA in D-PBS was perfused until D-PBS in the channel was completely replaced with 0.1% BSA perfusion media. A 50 μL pulse of 5×10^5^ cells/mL cell suspension in 0.1% BSA solution (total of 2.5×10^4^ cells) was then added to the inlet reservoir before initiating syringe withdraw at a flow rate appropriate for achieving the desired wall shear stress and beginning video acquisition of the experiment. The cell pulse was immediately followed by continuous perfusion of 0.1% BSA solution for the remainder of the 2 hr video acquisition. In select experiments, cell suspensions and perfusion medium contained 0.5, 5, or 25 U mL^-1^ heparin. Videos were acquired using NIS-Elements (Nikon, Melville, NY), with identical camera and software settings; exposure time was 0.281 μs, the frame rate was 25 frames per second, the objective magnification was 10x, the image size was 500 by 376 pixels, and the image was binned 2×2.

### Video analysis

Videos acquired during perfusion experiments were post-processed using a custom modified program based on the OpenCV Traffic Flow Analyzer (https://github.com/telescope7/TrafficFlowAnalysis). Briefly, the background was subtracted using a mask/weight of 0.0005, resulting in an approximate 80 s moving average. Frames were blurred by a factor of 13 and thresholded by a factor of 15. A Moving Object database stored detected contours > 5 μm in diameter for comparison to previously tracked objects. Using either a look ahead window or an overlapping object boundary analysis, detected objects were mapped to corresponding earlier locations of the same object. Object data was read to a file once the object could no longer be tracked or exited the field of view.

Free flow cell velocities were approximated as 529, 1059 and 1588 μm/s and rolling velocity thresholds were 125, 250, or 375 μm/s at 0.5, 1.0 and 1.5 dyn/cm^2^, respectively. Average velocity was calculated from the chamber length (L_channel_, 1.3 or 14 cm) divided by elution time (t_elution_) less the offset time (t_offset_, which accounts for the mean time cells spend in the settling feature before entering the main channel) (Eqn.1).

$$V_{avg}=\frac{L_{channel}}{\left(t_{elution}-t_{offset}\right)}$$ Eqn. 1

Percent binding time was calculated using Equation 2, which was derived from a mass balance and described previously [26], where V_free flow_ is the approximated free flow velocity, t_elution_ is the elution time, t_offset_ is the mean time spent in the settling feature, L_channel_ is the channel length, and V_inst_ is the instantaneous velocity in the field of view.

$$\%\;Binding\;time=\frac{\frac{\left(v_{free\;flow}\left(t_{elution}-t_{offset}\right)\right)-L_{channel}}{v_{free\;flow}-v_{isnt}}}{t_{elution}-t_{offset}}$$ Eqn. 2

### Statistical analysis

Data were analyzed using RStudio (version 3.3.1, Boston, MA) and statistics and plots were generated using GraphPad Prism 7 (GraphPad Software, Inc., La Jolla, CA). Data are displayed as either individual data points or as the mean ± SEM. Experiments were repeated in triplicate or more, save in select instances where duplicate measurements are reported. For determining the relationship between two variables, a linear regression was used and slopes determined to be statistically non-zero are indicated with an asterisk. Person's r was reported for determining the relationship between two measured variables. Two-way ANOVA with Bonferroni correction for multiple comparisons was used to compare the effect of selectin concentration, wall shear stress, or heparin concentration and cell type. One, two, three, and four symbols denotes statistical significance with p<0.05, p<0.01, p<0.001, and p<0.0001, respectively.

## Abbreviations

V_inst_: instantaneous velocity
V_avg_: average velocity
PMN polymorphonuclear leukocytes; PDMS: polydimethylsiloxane
D-PBS: Dulbecco's phosphate-buffered solution
BSA: bovine serum albumin
